# Immune checkpoint inhibitor monotherapy is associated with less cardiac toxicity than combination therapy

**DOI:** 10.1371/journal.pone.0272022

**Published:** 2022-11-01

**Authors:** Eugene B. Cone, Lorine Haeuser, Stephen W. Reese, Maya Marchese, David-Dan Nguyen, Junaid Nabi, Wesley H. Chou, Joachim Noldus, Rana R. McKay, Kerry Laing Kilbridge, Quoc-Dien Trinh

**Affiliations:** 1 Division of Urological Surgery and Center for Surgery and Public Health, Brigham and Women’s Hospital, Harvard Medical School, Boston, MA, United States of America; 2 Department of Urology and Neuro-Urology, Marien Hospital Herne, Ruhr-University Bochum, Germany; 3 Division of Urology, University of Toronto, Toronto, ON, Canada; 4 Division of Medical Oncology, San Diego, CA, United States of America; 5 Lank Center for Genitourinary Oncology, Dana-Farber Cancer Institute, Boston, MA, United States of America; Samsung Medical Center, REPUBLIC OF KOREA

## Abstract

**Background:**

Treatment options for many cancers include immune checkpoint inhibitor (ICI) monotherapy and combination therapy with impressive clinical benefit across cancers. We sought to define the comparative cardiac risks of ICI combination and monotherapy.

**Methods:**

We used VigiBase, the World Health Organization pharmacovigilance database, to identify cardiac ADRs (cADRs), such as carditis, heart failure, arrhythmia, myocardial infarction, and valvular dysfunction, related to ICI therapy. To explore possible relationships, we used the reporting odds ratio (ROR) as a proxy of relative risk. A lower bound of a 95% confidence interval of ROR > 1 reflects a disproportionality signal that more ADRs are observed than expected due to chance.

**Results:**

We found 2278 cADR for ICI monotherapy and 353 for ICI combination therapy. Combination therapy was associated with significantly higher odds of carditis (ROR 6.9, 95% CI: 5.6–8.3) versus ICI monotherapy (ROR 5.0, 95% CI: 4.6–5.4). Carditis in ICI combination therapy was fatal in 23.4% of reported ADRs, compared to 15.8% for ICI monotherapy (P = 0.058).

**Conclusions:**

Using validated pharmacovigilance methodology, we found increased odds of carditis for all ICI therapies, with the highest odds for combination therapy. Given the substantial risk of severe ADR and death, clinicians should consider these findings when prescribing checkpoint inhibitors.

## Introduction

One in five men and one in six women develop cancer during their lifetime, accounting for 18.1 million new cases and 9.6 million deaths globally in 2018 [1]. Although cancers vary widely in terms of their biology and interactions with the host, many rely on immune checkpoint pathways to suppress the immune system and evade host surveillance [2]. This commonality has recently been exploited by immune checkpoint inhibitors (ICI), which demonstrate impressive clinical benefit, especially in advanced and metastatic cancers [3].

ICIs block negative regulators of T-cell function on immune and tumor cells. The most extensively evaluated T-cell checkpoints are the cytotoxic T-lymphocyte antigen 4 (CTLA-4) and programmed cell death 1 (PD-1)/programmed cell death ligand 1 (PD-L1) pathways. ICIs are approved for various oncological indications, such as treatment of metastatic melanoma [4], non-small cell lung cancer (NSCLC) [5], renal cell carcinoma (RCC) [6], urothelial carcinoma [7], Hodgkin lymphoma [8], B-cell lymphoma [9], breast [10], head and neck [11], gastric [12], colorectal [13], and hepatocellular cancer [14]. National Comprehensive Cancer Network (NCCN) guidelines list both ICI monotherapy and combination therapy with differently acting ICIs as options for a variety of cancers [15–17].

ICIs can result in a wide range of adverse drug reactions (ADRs), with prior studies demonstrating a higher adverse events profile for patients treated with ICI combination therapy compared to monotherapy [18]. However, while cardiac toxicity is described with chemotherapy, the relative cardiac toxicity of ICIs in a real-world setting, especially in combination therapies, remains incompletely characterized [19]. Prior literature has reported cardiac ADRs including myocardial fibrosis [20], left ventricular dysfunction [21], cardiomyopathy [22], pericarditis [23], and myocarditis [24]. These events are uncommon, but the numbers of cardiac toxicity might be underestimated given the non-specific symptomatology, low incidence and the incomplete diagnostic testing [25]. Zamami et al. analyzed risk factors associated with ICI-related myocarditis and found higher odds in female patients, patients of older age (≥75 years) and the concomitant use of ICI treatment [26]. While Salem et al. found an over-representation of myocarditis, pericardial diseases and vasculitis cases in patients treated with ICI therapy [27], our study sought to directly compare the incidence of cardiotoxicity between ICI monotherapy and combination therapy. We therefore explored their comparative cardiac risk by analyzing a global pharmacovigilance database.

## Methods

### Study design and data source

We analyzed VigiBase, the World Health Organization (WHO) global database of individual case safety reports (ICSRs). Starting in 1968 with 10 contributing countries, VigiBase is now the largest ICSR database, with more than 130 participating countries and over 20 million reports of suspected ADRs, which are managed by the Uppsala Monitoring Centre (UMC). All reports until November 23, 2019 were analyzed. Reports to this pharmacovigilance database can be made by health professionals, pharmaceutical companies and patients themselves. The ICSRs are reviewed and analyzed locally and may lead to regulatory action [28, 29]. The study was approved by our Institutional Review Board Partners Human Research, reference number: 2019P000832. Consent to participate was waived because de-identified VigiBase reports were analyzed.

### Procedures

We identified cardiac ADRs (cADRs) reported in patients taking ipilimumab (CTLA-4 inhibitor), nivolumab (PD-1 inhibitor), pembrolizumab (PD-1 inhibitor), atezolizumab (PD-L1 inhibitor) or durvalumab (PD-L1 inhibitor) as ICI monotherapy (hereafter referred to as “monotherapy”) or combination therapy of ipilimumab and nivolumab (hereafter referred to as “combination therapy”), as a treatment for various solid and hematological cancers. For this purpose, we used the standardized Medical Dictionary for Regulatory Activities (MedDRA) terminology. Exploratory VigiBase inquiries revealed that the other guideline-recommended combination of pembrolizumab and axitinib, which was approved by the FDA in April 2019, did not occur with high enough frequency in the database (6 reports) to allow for valid analysis, so this combination was excluded. Every report had general administrative information (reporting date and country), patient demographic data (age, sex), drug-specific information (indication, dosage regimen, duration of therapy) and reported reactions (MedDRA classification terms, onset date, end date, seriousness, and final outcome). cADRs were sub-categorized in carditis (inflammatory cardiomyopathies, pericarditis, and myocarditis), heart failure (HF), arrhythmia, myocardial infarction (MI), new valvular dysfunction, and others.

### Statistical analysis

As there is no comparison group of patients who take ICI monotherapy or combination ICI therapy and do not experience the analyzed cADR in VigiBase, we used disproportionality analysis to study if cADRs were differentially reported with these regimens compared with cADRs reported in the entire database. In this case/non-case study, a significant association between the specific drug and the ADR is found if the proportion of a cADR is greater in patients exposed to the drug (case) than in patients exposed to any other drug in the database (non-case).

Disproportionality can be either shown using a reporting odds ratio (ROR) or an empirical Bayes estimator (EBE). The ROR is the pharmacovigilance equivalent of the Odds Ratio (OR), which is used in case/non-case studies as a measure of association. When the lower bound of the 95% confidence interval for the ROR is greater than 1, it indicates a disproportionality signal for the cADR of interest that is statistically greater than expected compared to all other non-cases [30–32]. A disadvantage of the ROR compared to an EBE is its large confidence interval due to significant sampling variability with low event counts.

The EBE, also a proxy of relative risk, considers a Poisson distribution for each cell count with an unknown true mean, fits prior and posterior distributions for the ratios and calculates posterior values, and can provide better estimates when event counts are low. We calculated the 5^th^ percentile value of the EBE for cADR as a screening cutoff for significance, then calculated a ROR for significant cases to define the reported risk in a more easily interpretable format [33].

Means and standard deviations (SD) were calculated, testing hypotheses with t-tests and chi squares for continuous and categorical variables. Analyses were performed using R (v3.6.1, RStudio).

## Results

We identified 2 631 cADRs from all VigiBase regions, spanning the years 2008–2019. Most of the reports originating from the Americas (48.3%) and Europe (36.5%) and most were reported outside of a clinical trial (59.1%). Of these, 2 278 reports were associated with ICI monotherapy and 353 with combination therapy. Almost all cADRs were classified as severe (88.3% in combination therapy and 84.1% in monotherapy, p = 0.059), which is defined in VigiBase as life-threatening, leading to disability, requiring hospitalization, or causing death. Fatal outcomes for overall cADR occurred in 18.9% and 16.4% of ICI monotherapy and combination therapy reports respectively (p = 0.23). Full details of the clinicodemographics associated with cADR for monotherapy and combination therapy are available in Tables 1 and 2.

**Table 1:** Characteristics of cardiac ADRs associated with immune checkpoint inhibitor therapy in Vigibase (last accessed 11/23/2019)^+^*.

Adverse Drug Reaction	Monotherapy (n = 2278)	Combination Therapy (n = 353)	p-value	Total (n = 2631)
**Region reporting**			<0.001	
Americas	1 012 (44.4)	259 (73.4)		1 271 (48.3)
Europe	877 (38.5)	83 (23.5)		960 (36.5)
Australia	368 (16.2)	11 (3.1)		379 (14.4)
Asia	12 (0.5)	0 (0.0)		12 (0.5)
Africa	3 (0.1)	0 (0.0)		3 (0.1)
Eastern Mediterranean	6 (0.3)	0 (0.0)		6 (0.2)
**Reported outside clinical trial**	1420 (62.3)	134 (38.0)	<0.001	1554 (59.1)
**Reported by non-healthcare worker**	283 (12.5)	65 (18.5)	0.003	348 (13.3)
**Age at onset (years)**			0.01	
75 or older	420 (18.4)	50 (14.2)		470 (17.9)
65–74	623 (27.3)	105 (29.7)		728 (27.7)
45–64	526 (23.1)	86 (24.4)		612 (23.3)
<45	84 (3.7)	20 (5.6)		104 (4.0)
unknown	625 (27.4)	92 (26.1)	0.20	717 (27.3)
**Male sex**	1 431 (66.6)	194 (61.4)	0.40	1 625 (65.9)
**Suspected drugs**				
Only drug of interest	1 786 (78.4)	288 (81.6)		2 074 (78.8)
1 other drug	282 (12.4)	37 (10.5)		319 (12.1)
2+ other drugs	210 (9.2)	28 (7.9)	0.37	238 (9.0)
**Time to ADR (days): mean (SD)**	83.4 (133.2)	100.1 (167)	0.059	84.4 (135.3)
**Severe ADR** ^**^**^	1841 (84.1)	308 (88.3)	0.23	2149 (84.7)
**Death as outcome**	430 (18.9)	58 (16.4)		488 (18.5)

+ Values are reported as n (%) unless otherwise indicated; Hypotheses testing with t-tests for continuous and chi squares for categorical variables

* Percentage ratios may vary by category owing to missing data (i.e., 1 event may account for a different column percent in Region Reporting vs Time to ADR)

^ Defined in VigiBase as life-threatening, leading to persistent or significant disability, birth defect, congenital anomaly, or to any other medically important condition, requiring hospitalization or causing death

**Table 2:** Number of cardiac events associated with immune checkpoint inhibitor monotherapy or combination therapy by indication (total n = 2 631, reporting n (%)).

Drug Indication	Monotherapy	Combination Therapy	Total
**Lung cancer**	967 (47.1)	38 (11.1)	1 005 (41.9)
**Skin cancer**	356 (17.3)	171 (50)	527 (21.9)
**Kidney cancer**	179 (8.7)	27 (7.9)	206 (8.6)
**Genitourinary cancer**	129 (6.3)	11 (3.3)	140 (5.9)
**Hematological cancer**	66 (3.2)	12 (3.5)	78 (3.3)
**Head and Neck**	57 (2.8)	2 (0.6)	59 (2.5)
**Gastrointestinal cancer**	50 (2.4)	18 (5.2)	68 (3.0)
**Breast**	25 (1.2)	0 (0.0)	25 (1.0)
**Other**	226 (11)	63 (18.5)	289 (12.1)

cADRs were reported 353 times for combination therapy and 2 278 times for monotherapy, which was in line with expected counts based on all-other-cause rates in VigiBase. The most common cADRs were arrhythmia (851/2631) then carditis (737/2631), MI (463/2631) and HF (338/2631). There were 630 episodes of carditis reported in ICI montherapy, which was 390% more than the expected count of 128.6 (ROR 4.96, 95% CI 4.59–5.37). However, 107 episodes of carditis were reported in ICI combination therapy, 577% more than the expected count of 15.8 (ROR 6.85, 95% CI 5.66–8.28). There was no significant difference in the proportion of episodes of carditis being classified as severe between combination therapy and monotherapy, which was reported in 86% and 81.8% of patients respectively (p = 0.37). Combination therapy was fatal for 23.4% of the reported carditis events, compared to a 15.8% fatality rate for monotherapy (p = 0.058). The full results for all studied reactions in ICI monotherapy and combination therapy are shown in Table 3.

**Table 3:** Number of reports, expected count, empirical Bayes estimator (EBE), and reporting odds ratio (ROR) for cardiac adverse drug reactions in patients receiving immune checkpoint inhibitor monotherapy or combination therapy.

	Count	Expected Count	Empirical Bayes Estimator	Reporting Odds Ratio (95% CI)
**Any cardiac event**				
Monotherapy	2 278	3 350	0.66	NA
Combination Therapy	353	410	0.79	NA
**Heart Failure**				
Monotherapy	313	333.2	0.85	NA
Combination Therapy	25	40.8	0.43	NA
**Myocardial Infarction**				
Monotherapy	415	753.6	0.51	NA
Combination Therapy	48	92.3	0.40	NA
**Carditis**				
Monotherapy	630	128.6	**4.57**	**4.96 (4.59–5.37)**
Combination Therapy	107	15.8	**5.70**	**6.85 (5.66–8.28)**
**Arrhythmia**				
Monotherapy	701	1 105.0	0.60	NA
Combination Therapy	150	135.4	0.96	NA
**Valvular Dysfunction**				
Monotherapy	9	109.9	0.05	NA
Combination Therapy	2	13.5	0.05	NA
**Other**				
Monotherapy	210	912	0.20	NA
Combination Therapy	21	111.7	0.13	NA

EBE reports the lower (5th percentile) bounds of the posterior distribution of odds. ADRs with concordant significant findings for EBE and ROR in bold. NA = not applicable.

**Fig 1:**
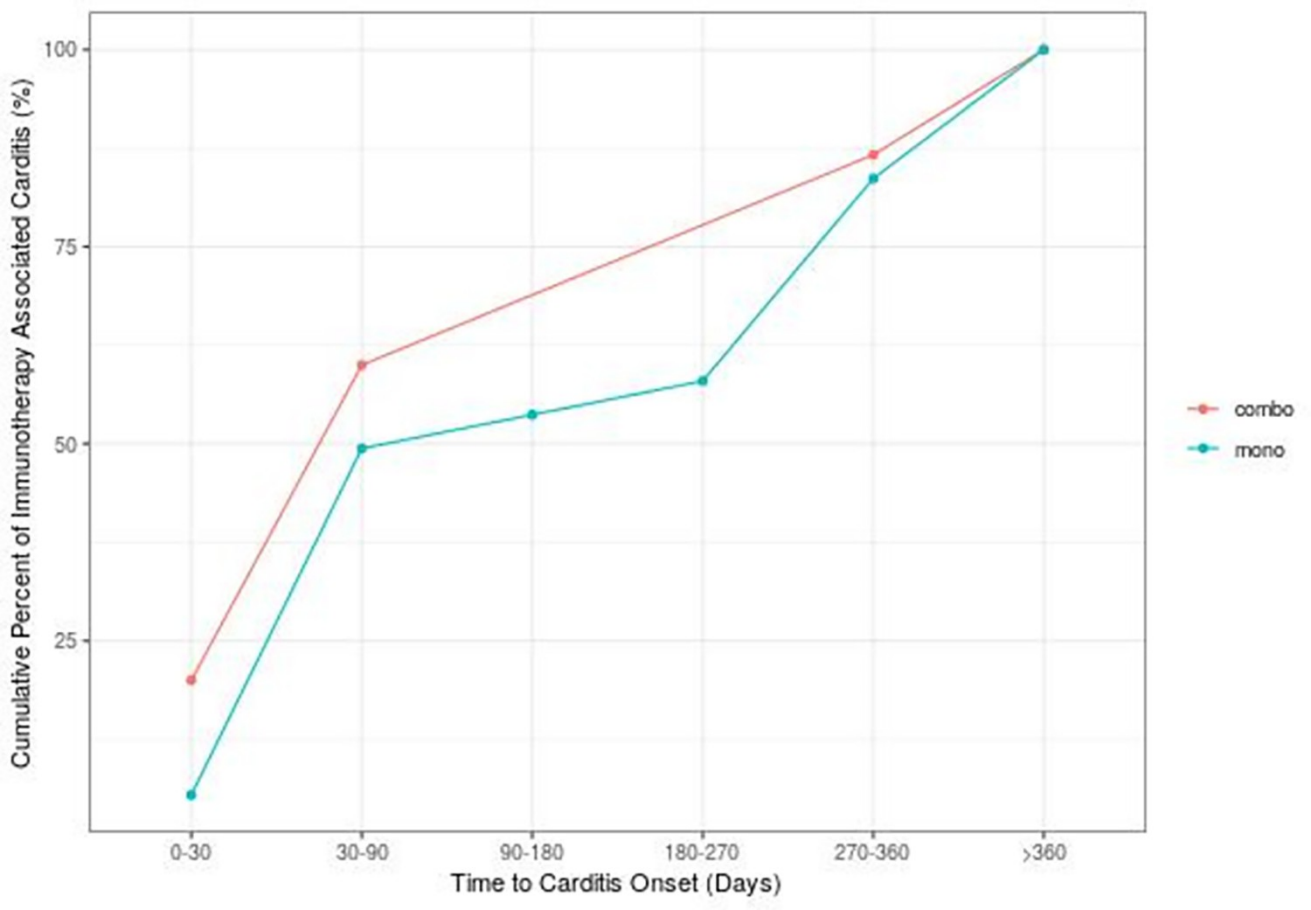
Cumulative incidence of time to carditis onset from initiation of immune checkpoint inhibitor monotherapy (turquoise) or combination therapy (pink).

Time to onset of carditis was earlier for combination therapy than for monotherapy. While 20% of all the carditis events in patients treated with combination therapy occurred in the first month and 60% within 90 days, only 5% of the carditis events occurred for monotherapy in the first month and 50% within 90 days. Cumulative incidence curves of time to carditis onset are displayed in Fig 1, with 13.4% of combination therapy patients and 14.4% of monotherapy patients experiencing reactions greater than 1 year after initiation of therapy (p = 0.76).

## Discussion

Analyzing the world’s largest pharmacovigilance database, we found higher reported risks for developing carditis (inflammatory cardiomyopathies, pericarditis, and myocarditis) in patients treated with ICI combination therapy compared to monotherapy. These events were severe in most cases (86% and 81.8% for combination therapy and monotherapy respectively), and more often fatal for combination therapy versus monotherapy (23.4% vs. 15.8% respectively). The majority of events occurred in the first 90 days of combination ICI therapy (60%) which contrasts to a majority occurring after greater than 180 days of administration for ICI monotherapy.

A challenge of administering ICI therapy is the occurrence of immune-related adverse events (irAEs), which can require cessation of the treatment in up to 40% of patients [18, 34]. Particularly pertinent to our study is myocarditis, a rare but severe irAE with an incidence of 0.04–1.14% but a mortality rate of 25–50% [35, 36]. The idea of ICI combination therapy is to overcome resistance and broaden the clinical utility of ICI by enhancing major discriminatory functions of the immune system altered by malignancies: antigenicity, adjuvanticity, and homeostatic feedback inhibition [37]. Combination therapy with ipilimumab and nivolumab shows promising results in patients with many solid tumors as well as lymphoma, but as seen in our study and prior work, it can be associated with an even further increased risk of irAEs as compared to monotherapy. Johnson et al. interrogated a safety database with over 20 000 patients undergoing nivolumab +/- ipilimumab therapy, and found that patients who received combination therapy experienced significantly more frequent and severe myocarditis compared to nivolumab monotherapy (0.3% vs. 0.06%), although their event rate was low with 5 vs. 1 fatal events [24].

It is unclear by what mechanism ICI-related myocarditis occurs. Of note, prior studies have shown that PD-1 and PD-L1 are constitutively expressed in mouse and human cardiomyocytes [38] and that CTLA-4 and PD-1 deletions are associated with autoimmune myocarditis in mice [39–41]. Theories to explain ICI-related myocarditis have largely centered around a common or homologous antigen between the cardiomyocyte and tumor being targeted by T cells, although there have been case studies without expected lymphocytic infiltration in the setting of nivolumab-induced myocarditis [25].

### Comorbidities

It remains unexplored whether increased comorbidity of an organ system raises its risk of related irAEs. Although autoimmune disorders have been associated with increased risk of irAEs in general [42, 43], it is unclear whether patients with pre-existing cardiac conditions are at increased risk of cardiac irAEs. In a prior report on two fatal cases where fulminant myocarditis and myositis occurred after the first dose of combination therapy, both patients had a history of hypertension, but no other cardiac risk factors [24]. Previous studies proved that about 50% of the patients presenting with myocarditis due to ICI therapy, develop concurrent irAEs, such as myositis and myasthenia gravis [44, 45]. Our research confirms the increased cardiac risk profile of both ICI combination therapy and monotherapy, but as with so much of immunooncology many questions remain.

### Onset

Carditis events can occur after only one or two doses of ICI, and have been observed to have an early median onset time of 27 days [46], although as seen in our data the onset can occur significantly later than that with irAEs observed over a year after initiation in some cases. We observed that the toxicity of combination ICI therapy occurs primarily within the first 90 days aligns. In combination with the fact that combination therapy with the CTLA-4 inhibitor ipilimumab is typically provided for four doses at the initiation of ICI therapy, this suggests that the additional cardiac toxicity of combination likely diminishes over time. However, more granular study would be required to tease out this subtlety.

### Severity

Our study as well as prior literature have shown that cADRs are associated with high fatality rates. However, cADRs span a wide spectrum of symptoms, ranging from abnormal cardiac biomarker testing without symptoms to severe decompensation [47]. This combined with the lack of standardized guidelines by which to perform cardiac monitoring, likely mean that the reporting of cardiotoxic events is biased towards severe events. Diagnostic testing should aim not only to confirm the diagnosis of carditis, but also rule out other more common cardiac causes of the clinical manifestations described above, such as acute coronary syndrome. Nevertheless, the severity of these cases shows that while uncommon, further research on diagnostic and monitoring strategies for these complications is likely warranted to ensure early treatment. Thus far, treatment has largely involved cessation of the ICI and glucocorticoids, although various other modalities such as intravenous immunoglobulin, infliximab, and abatacept have been trialed [36].

### Limitations

There are several limitations that need to be considered for this analysis. First, due to the variety of reporting sources within VigiBase, we are limited in our ability to verify the correctness of clinical, laboratory or radiological findings that lead to a specific diagnosis or the completeness of drug dose and concomitant drugs, age, comorbidities, and time to onset. Furthermore, the assessment of combination therapy may be limited by reporting, especially for reports by patients themselves who might not be aware of taking two different immune checkpoint inhibitors. As with all pharmacovigilance research, the likelihood that the reported event is due to the drug in question can differ between the reports. While some countries report only ADRs with a possible causal relationship between drug and event, others collect any adverse event, even if not considered drug related. However, more permissive reporting weakens any associations observed, has a significant effect on all-other-cause rates, and would bias away from our conclusions [48]. It is likely that there are more ADRs than reported to the national centers for inclusion to VigiBase, which should not bias results in the absence of unbalanced reporting, but can limit power. In addition, the exact denominator of patients taking pembrolizumab, ipilimumab, nivolumab, atezolizumab, durvalumab or a combination therapy of ipilimumab and nivolumab is unknown. This is a limitation inherent to all pharmacovigilance research, requiring the use of disproportionality analysis and signal detection techniques [49, 50]. Lastly, we did not stratify by dose of ICI administered, so we were unable to assess dose effects or relative contributions of higher/lower doses by disease state.

## Conclusions

Using validated pharmacovigilance techniques, we identified significantly increased reported odds of inflammatory cardiomyopathies, pericarditis, and myocarditis for all evaluated ICI, with the highest odds for combination therapy with ipilimumab and nivolumab. These increased risks should be incorporated when considering monotherapy or combination therapy regimens in the care of cancer patients. Clinicians should consider these findings and emphasize prompt diagnosis and management of these cADRs, counseling patients about side effects of the ICI therapy and the importance of disclosing early symptoms to the provider.

## Supporting information

S1 Questionnaire (DOCX)

## Abbreviations

ADR: Adverse Drug Reaction
cADR: Cardiac Adverse Drug Reaction
CI: Confidence Interval
CTLA-4: Cytotoxic T-lymphocyte Antigen 4
EBE: Empirical Bayes Estimator
HF: Heart Failure
irAE: Immune-Related Adverse Event
ICI: Immune Checkpoint Inhibitor
ICSR: Individual Case Safety Report
MedDRA: Medical Dictionary for Regulatory Activities
MI: Myocardial Infarction
NCCN: National Comprehensive Cancer Network
NSCLC: Non-Small Cell Lung Cancer
PD-1: Programmed Cell Death 1
PD-L1: Programmed Cell Death Ligand 1
RCC: Renal Cell Carcinoma
ROR: Reporting Odds Ratio
SD: Standard Deviation
WHO: World Health Organization
